# Integrating diagnostics for elimination

**DOI:** 10.1186/1475-2875-11-S1-O27

**Published:** 2012-10-15

**Authors:** Chris J Drakelev

**Affiliations:** 1 Immunology & Infection Department, London School of Hygiene & Tropical Medicine, London, WC1E 7HT, UK

## 

There are increasing reports of declining malaria prevalence in a number of African countries. This presents a new set of challenges for accurate identification and enumeration of the parasite burden. This is not solely related to generating estimates of malaria with sufficient precision (which may be poor for entomological measures for example) but to the realisation that these measures constitute an important component of surveillance. Pro-and reactive surveillance will be required to maintain the gains that have been made by control programmes by better targeting of resources to areas of sustained transmission which are likely constitute the reservoirs for infection. Surveillance is also an integral component of elimination campaigns.

To facilitate surveillance and monitoring, new tools for the detection of infection and exposure to infection will be needed. Molecular amplification assays such as PCR or LAMP will allow identification of sub-microscopic infections that are likely to be the reservoir of infection at low transmission. Serological measures detect antibodies to malaria parasites that reflect exposure to infection and can be performed on large sample numbers. It is likely that these methodologies will need to be used both in conjunction to capture as many likely infections as possible but also separately as the scale and sensitivity of screening change.

This presentation will provide further rationale for integrating these approaches, show current examples of their utility in low endemicity settings and discuss options for subsequent development of this approach.

